# Epidermal Growth Factor Modulates Palmitic Acid-Induced Inflammatory and Lipid Signaling Pathways in SZ95 Sebocytes

**DOI:** 10.3389/fimmu.2021.600017

**Published:** 2021-05-06

**Authors:** Dániel Törőcsik, Fruzsina Fazekas, Szilárd Póliska, Andrea Gregus, Eszter Anna Janka, Katalin Dull, Andrea Szegedi, Christos C. Zouboulis, Dóra Kovács

**Affiliations:** ^1^ Department of Dermatology, Faculty of Medicine, University of Debrecen, Debrecen, Hungary; ^2^ Genomic Medicine and Bioinformatic Core Facility, Department of Biochemistry and Molecular Biology, Faculty of Medicine, University of Debrecen, Debrecen, Hungary; ^3^ Division of Dermatological Allergology, Faculty of Medicine, University of Debrecen, Debrecen, Hungary; ^4^ Departments of Dermatology, Venereology, Allergology and Immunology, Dessau Medical Center, Brandenburg Medical School Theodor Fontane and Faculty of Health Sciences Brandenburg, Dessau, Germany

**Keywords:** palmitic acid, sebum, EGF, sebocytes, acne

## Abstract

Epidermal growth factor (EGF) acts as a paracrine and autocrine mediator of cell proliferation and differentiation in various types of epithelial cells, such as sebocytes, which produce the lipid-rich sebum to moisturize the skin. However, sebum lipids *via* direct contact and by penetrating through the epidermis may have regulatory roles on epidermal and dermal cells as well. As EGF receptor (EGFR) is expressed throughout the proliferating and the lipid-producing layers of sebaceous glands (SGs) in healthy and acne-involved skin, we investigated the effect of EGF on SZ95 sebocytes and how it may alter the changes induced by palmitic acid (PA), a major sebum component with bioactive roles. We found that EGF is not only a potent stimulator of sebocyte proliferation, but also induces the secretion of interleukin (IL)6 and down-regulates the expression of genes involved in steroid and retinoid metabolism. Importantly, when applied in combination with PA, the PA-induced lipid accumulation was decreased and the cells secreted increased IL6 levels. Functional clustering of the differentially regulated genes in SZ95 sebocytes treated with EGF, PA or co-treated with EGF+PA further confirmed that EGF may be a potent inducer of hyperproliferative/inflammatory pathways (IL1 signaling), an effect being more pronounced in the presence of PA. However, while a group of inflammatory genes was up-regulated significantly in EGF+PA co-treated sebocytes, PA treatment in the absence of EGF, regulated genes only related to cell homeostasis. Meta-analysis of the gene expression profiles of whole acne tissue samples and EGF- and EGF+PA -treated SZ95 sebocytes showed that the EGF+PA co-activation of sebocytes may also have implications in disease. Altogether, our results reveal that PA-induced lipid accumulation and inflammation can be modulated by EGF in sebocytes, which also highlights the need for system biological approaches to better understand sebaceous (immuno)biology.

## Introduction

Sebaceous glands (SGs) form together with the hair follicle the pilosebaceous unit, with a primary function to produce sebum to cover and lubricate the hair and the skin (1). Several studies, however, suggested, that sebum lipids are not only moisturizers, but may have additional biological functions (2–4). While PA together with sapienic/palmitoleic (5) and oleic acids have antimicrobial activities (6, 7), other lipids, such as squalene, are known to be ultraviolet (UV) protective (8–10). Moreover, the findings that i., SG-rich skin had a distinct immune milieu compared to SG-poor skin (11) ii., a dynamic change both in the amount and the ratio of sebum lipid fractions can be observed in acne (12, 13), the primary disease associated with the inflammation of the pilosebaceous unit affecting nearly 90% of teenagers in the Western societies (14), and iii., sebaceous lipids may penetrate through the epidermis (15, 16) or even directly infiltrate the dermis when the pilosebaceous unit is destroyed in severe acne, altogether suggest that sebocytes may contribute to the dermal microenvironment with complex regulatory functions on various cell types (17). In previous own studies we showed that besides promoting T helper (Th) 17 cell differentiation *via* secreted proteins (18), sebocytes are able to alter gene and protein expression through sebaceous lipids in normal human keratinocytes and HaCaT cells (19), while in human monocyte-derived macrophages each lipid had a selective immunomodulatory effect (16). Supporting a key position for PA in shaping the inflammatory environment, we confirmed that PA is a more potent stimulator of interleukin (IL)1 beta (IL1B) and tumor necrosis factor alpha (TNFA) cytokine production than *Propionibacterium acnes (P. acnes)*, the commensal bacterium which has been associated with acne, in *in vitro* differentiated monocyte-derived macrophages (16). Moreover, PA treatment was shown to increase the secretion of IL6 and IL8 in SZ95 sebocytes (20), cytokines that contribute to inflammation in acne lesions (21).

Of the various factors known to affect sebocyte function (22), such as hormones (23–30), neuropeptides (31–33) and Toll-like receptor (TLR) ligands (34–36), EGF has a central role, underpinned by the pioneering finding that it is essential to maintain sebocytes in culture (37) and that its receptor (EGFR), a member of the ErbB family of receptor tyrosine kinases (38) also known as ERBB1 or HER1, had an increased density in the peripheral layer of the glands, where proliferation dominates over differentiation and lipid accumulation (39–42). Later studies revealed that although EGF activation or overexpression in mouse leads to enlarged SGs with hyperproliferating sebocytes and increased lipogenesis (43, 44), in human sebocytes EGF treatment resulted in an increased proliferation and reduced lipogenesis (24). This was further supported by increased lipid accumulation resulting through small interfering RNA (siRNA)-mediated down-regulation of EGFR in SZ95 sebocytes (45). Moreover, a possible interaction with testosterone also suggested that at the time of puberty, EGF may lead to SG hypertrophy and thus be involved in the development of acne (46). Importantly, EGF as well as other EGFR ligands, such as transforming growth factor alpha (TGFA), epigen, epiregulin, amphiregulin, betacellulin, and heparin-binding EGF are found in detectable amounts in the serum. More importantly, they can also be produced by different skin cell types, such as keratinocytes, fibroblasts, and sebocytes (47–49), suggesting that EGFR activation in the skin may happen both in a paracrine and an autocrine manner mediating signals of local as well as of systemic origin.

While the postulate that EGF signaling in humans may be crucial in balancing sebocytes between proliferation and lipogenesis grants EGF a prime position in sebaceous biology, there is still little data available on how it may alter the inflammation signaling in sebocytes. The findings that EGFR inhibitor-treated SZ95 sebocytes exhibit increased expression levels of the inflammatory cytokines IL6, IL8 and TNFA, but not of IL1 alpha (IL1A) (50) suggested that EGF is also involved in the regulation of inflammation, but more extensive studies, such as genome wide analyses, are needed to reveal the complex changes in which EGF may be involved. Thus, in this study we addressed the question of how EGF itself and in combination with PA, a (patho)physiologically relevant lipid with both differentiating as well as inflammatory properties (6, 16, 51, 52), could interact to modify phenotype, gene expression and inflammation in human sebocytes.

## Materials and Methods

### Cell Culture and Treatments

**Figure 1:**
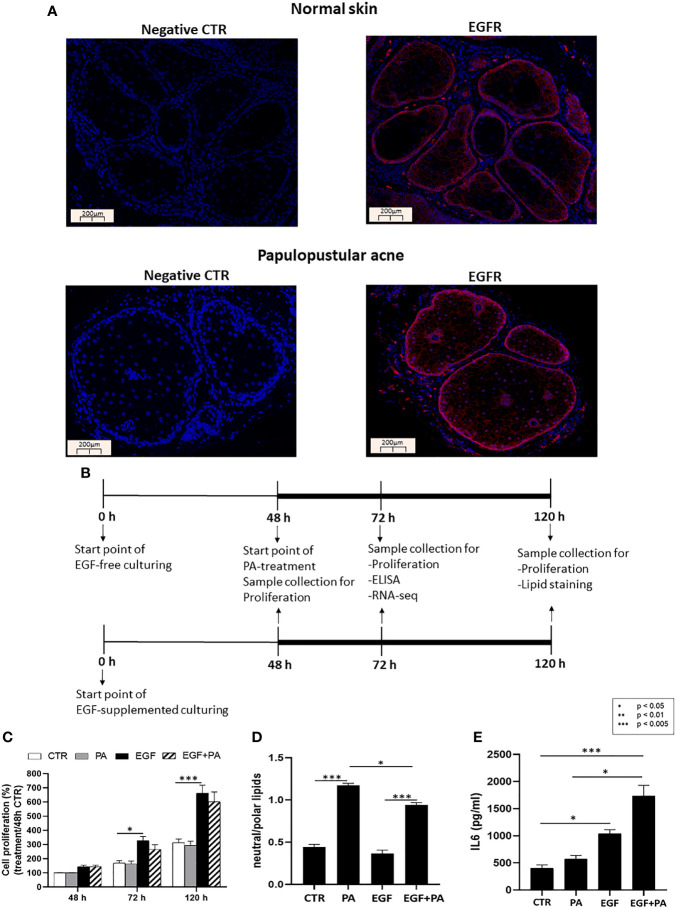
EGFR is expressed in sebaceous glands of healthy and acne involved skin and EGF modulates proliferation, lipid production and inflammation in SZ95 sebocytes. **(A)** FFPE tissue samples were stained with an anti-EGFR rabbit polyclonal antibody as described in Materials and Methods. Note that similar staining intensities are observed in normal and in papulopustular acne skin samples, localizing to both the proliferating as well as the lipid producing layers of sebaceous glands. Negative controls were processed without using primary antibody. Slides were mounted with Vectashield mounting medium with DAPI. Scale bar = 200 µm. **(B)** SZ95 sebocytes were cultured in the presence or absence of EGF. At 48 hours 150 µmol/L PA or vehicle was added to the medium and SZ95 sebocytes were cultured for further 24 and 72 hours (marked as 72 h and 120 h). Samples were collected, processed, and measured as described in Materials and Methods at the relevant timepoints. **(C)** Sulforhodamine B assay was used as described in Materials and Methods to detect the rate of proliferating sebocytes cultured in the presence or absence of EGF for 48, 72 and 120 hours and treated with 150 µmol/L PA, at 48 hours timepoint. EGF significantly induced cell proliferation at 72 and 120 hours, whereas proliferation was tendentiously decreased after co-treatment with PA. Samples from three subcultures of SZ95 sebocytes were used for the experiments and three independent measurements were performed. SRB data were normalized to the absorbance of the 48 hours CTR samples which was taken for 100%. Two-way ANOVA and Tukey post-hoc tests were used in the analysis of SRB data. All data are presented as mean ± SEM. *p < 0.5, ***p < 0.005. **(D)** AdipoRed staining used to detect polar and neutral lipids as described in Materials and Methods in SZ95 sebocytes cultured in the presence or absence of EGF and treated with 150 µmol/L PA for 72 hours showed that EGF decreased PA-induced lipogenesis. Amount of neutral lipids are normalized with polar lipids, which correlate with cell numbers. Results are expressed as average of three independent measurements using samples from three subcultures of SZ95 sebocytes. Kruskal-Wallis test and Dunn post-hoc tests were used in the analysis of data. All data are presented as mean ± SEM. *p < 0.05, ***p < 0.005. **(E)** Supernatants from SZ95 sebocytes cultured in the presence or absence of EGF and treated with 150 µmol/L PA for 24 hours were used to determine the levels of IL6 by ELISA as described in Materials and Methods. Results are expressed as average of protein concentrations (pg/mL) of five independent measurements using samples from three subcultures of SZ95 sebocytes. Kruskal-Wallis test and Dunn post-hoc tests were used in the analysis of ELISA data. All data are presented as mean ± SEM. *p < 0.05, ***p < 0.005.

Immortalized human SZ95 sebocytes (53) were cultured at 37°C in a humidified atmosphere containing 5% (v/v) CO_2_ in Sebomed^®^ Basal Medium (Cat. No.: F8205, Biochrom, Cambridge, UK) supplemented with 10% fetal bovine serum (FBS) (BioSera, Nuaillé, France), 1 mmol/L CaCl_2_, 500U/ml penicillin, 0.5 mg/ml streptomycin (Sigma-Aldrich, St. Louis, MO, USA) and 5 ng/ml EGF (dissolved in distilled water) (Sigma-Aldrich). For experiments, cells were pre-conditioned in EGF-free or in EGF-supplemented medium for 48 hours, then passaged and re-plated keeping the conditions of EGF depletion or supplementation. Re-plated sebocytes were treated with 150 µmol/L PA (Cat. no.: P0500, Sigma-Aldrich) dissolved in ethanol:dimethyl sulfoxide (DMSO) in 1:1 ratio, heated at 37°C for 10 minutes and mixed vigorously. The applied PA dose was previously determined (**Supplementary Figure 1**). Untreated cells were incubated with ethanol:DMSO in 1:1 ratio (vehicle control). Cell treatment conditions were the following: CTR = EGF-free vehicle control; EGF = EGF-supplemented and treated with vehicle; PA = PA-treatment in absence of EGF; EGF+PA = PA-treatment in presence of EGF. SZ95 sebocytes from three subcultures were used for all experiments. An overview of our experimental setup is shown in **Figure 1B**, where we indicate the culturing conditions and the timepoints for sample collections.

### Immunofluorescence Staining

Anonymized formalin-fixed and paraffin embedded (FFPE) sections of human tissue samples of lesional skin from the back of patients with papulopustular acne and from the back of healthy individuals were acquired from the archive of the Department of Dermatology, University of Debrecen with the approval of the Regional and Institutional Ethics Committee. 3 µm thick FFPE sections were deparaffinized and rehydrated. For antigen retrieval, the slides were treated with Tris- ethylenediaminetetraacetic acid (EDTA) buffer (10mM Tris Base, 1mM EDTA solution, 0.05% Tween 20, pH 9.0) in boiling pressure cooker (120°C) for 25 minutes and incubated with 5% bovine serum albumin (BSA) dissolved in phosphate buffered saline (PBS) to reduce non-specific binding. Tissue sections were incubated with polyclonal rabbit anti-human EGFR antibody (Cat. no.: sc-03; Santa Cruz, Dallas, TX, USA diluted in 5% BSA-PBS at a 1:50 dilution) in a humidity chamber at 4°C for overnight, while in the negative controls no primary antibody was used. Goat anti-rabbit IgG Alexa Fluor 555 secondary antibody (Thermo Fisher Scientific, Waltham, MA, USA) was used in accordance with the manufacturer’s instructions. Slides were mounted with Vectashield mounting medium with 4′,6-diamidino-2-phenylindole (DAPI) (Vector Laboratories, Burlingame, California, USA) and images were acquired with a Leica DM2000 LED microscope (Leica Microsystems, Wetzlar, Germany) connected to an Olympus DP74 camera (Shinjuku-ku, Tokyo, Japan).

### Sulforhodamine B (SRB) Colorimetric Proliferation Assay

To detect the proliferation rate, samples were collected at time of re-plating at 48 hours, then at 24 and 72 hours after PA treatment (marked as 48, 72 and 120 h in **Figure 1C**) as described previously. After removing the culture medium, cells were fixed with cold 10% trichloroacetic acid (TCA) overnight at 4°C and subsequently washed four times with tap water and completely dried. 50 µl 0.04% SRB (dissolved in 1% acetic acid) (Sigma-Aldrich) was added to each well and plates were incubated at room temperature for 1 hour. To remove unbound dye, plates were rinsed four times with 1% acetic acid. To solubilize the protein-bound dye, 50 µl of 10 mmol/L Tris base solution (pH 10.5) was added to each well and plates were shaken on an orbital shaker for 10 minutes. The optical density (OD) was measured at 510 nm in an Epoch microplate spectrophotometer (BioTek, Winooski, VT, USA). SRB absorbance value of the 48 hours CTR sample was taken for 100% and was used for normalization in all measurements. SZ95 sebocytes from three subcultures were used, samples were measured in five replicates from three independent experiments.

### Lipid Staining

To detect intracellular lipids, SZ95 sebocytes were cultured and treated with PA as described above for 72 hours. Cells were then washed with PBS and stained with AdipoRed (Lonza, Basel, Switzerland) according to the manufacturer’s instructions. Neutral lipids were detected with excitation at 485 nm and emission at 565 nm, while polar lipids with excitation at 540 nm and emission at 620 nm using a Tecan Spark 20M fluorometer (Tecan Trading AG, Männedorf, Switzerland). Arbitrary fluorescent unit (AFU) score ratios of neutral lipids and of polar lipids are presented. SZ95 sebocytes from three subcultures were used, samples were measured in five replicates in three independent experiments.

### Enzyme-Linked Immunosorbent Assay (ELISA)

To measure the secretion of IL6 protein, SZ95 sebocytes were cultured as described above. Supernatants were collected at 24 hours after PA treatment, aliquoted and stored at -20°C until further analyses. IL6 protein levels were measured using ELISA Duoset (Cat. No.: DY206, R&D Systems, Minneapolis, MN, USA) according to the manufacturer’s instructions. 3,3′,5,5′- Tetramethylbenzidine (TMB) (Sigma-Aldrich) chromogenic substrate was used as visualizing reagent and the reaction was stopped with 1mol/L H_2_SO_4._ Optical density was measured in an Epoch microplate spectrophotometer (BioTek) at 450 nm wavelength. SZ95 sebocytes from three subcultures were used, samples were measured in duplicates in five independent experiments.

### Determination of mRNA Levels

SZ95 sebocytes were cultured for 24 hours after PA treatment, as previously described. Total RNA was isolated using TRI Reagent (MRC, Cincinnati, OH, USA) according to the manufacturer’s protocol. Total RNA sample quality was checked on Agilent BioAnalyzer using Eukaryotic Total RNA Nano Kit according to manufacturer’s protocol (Agilent, St. Laurent, Quebec, Canada). Samples with RNA integrity number (RIN) value >7 were accepted for library preparation process. RNA-Seq libraries were prepared from total RNA using Ultra II RNA Sample Prep kit (New England BioLabs, Évry-Courcouronnes, France) according to the manufacturer’s protocol. Briefly, poly-A RNAs were captured by oligo-dT conjugated magnetic beads, then the mRNAs were eluted and fragmented at 94°C. First strand cDNA was generated by random priming reverse transcription and after second strand synthesis step double stranded cDNA was generated. After repairing ends, A-tailing and adapter ligation steps adapter ligated fragments were amplified in enrichment PCR and finally sequencing libraries were generated. Sequencing run was executed on Illumina NextSeq500 instrument (Illumina, San Diego, CA) using single-end 75 cycles sequencing.

### RNA-Seq Data Analysis

Raw sequencing data (fastq) was aligned to human reference genome version GRCh38 using HISAT2 algorithm and BAM files were generated. Downstream analysis was performed using StrandNGS software (version 2.8, build 230243; Strand Life Sciences, Bangalore, India). BAM files were imported into the software, DESeq algorithm was used for normalization and normalized expression data were used for statistical analysis. Biological process, Reactome and KEGG pathway analyses were performed using the Cytoscape 3.7.1 software with the ClueGO v2.5.4 plug-in (54). Results are expressed as average mean values of three samples from subcultures of SZ95 sebocytes. Heat maps displaying the replicates separately are provided as a supplement to support the heat maps in the figures that show mean average values (**Supplementary Figures 2-11**). Principal component analysis (PCA) shows the possible batch-to-batch variation in the dataset (**Supplementary Figure 12**). Gene expression data of differentially up- and down-regulated genes were filtered with 2-fold change. RNA-Seq data are available in Sequence Read Archive (SRA) database, under accession number: PRJNA646337 (https://www.ncbi.nlm.nih.gov/sra/PRJNA646337).

### Statistical analysis

All data are presented as mean ± SEM. The normality of the population was determined using the Shapiro-Wilk test. One-way ANOVA and Tukey post-hoc tests were used for the analysis of RNA-seq data. For cell proliferation experiments, two-way ANOVA supplemented with Tukey post-hoc test was used. Lipid assay and ELISA experiments were analyzed by Kruskal-Wallis test and Dunn post-hoc test. Differences by p<0.05 values were considered statistically significant.

## Results

### EGFR Is Expressed in the Basal Layer as Well as in the Lipid Producing Cells of Sebaceous Glands

To provide a biological relevance for our hypothesis that EGF and lipids may orchestrate the functions of sebocytes both under physiological as well as pathological conditions, we first performed immunofluorescence staining with a specific EGFR antibody in human skin samples of healthy individuals (normal skin) and of patients with papulopustular acne. We confirmed the results of Nanney et al. (40) that the expression of EGFR is not restricted to the basal proliferating layer (39) but, although with a weaker signal, is also detected in the lipid-producing sebocytes of healthy as well as acne-involved skin (**Figure 1A**).

### EGF Modulates Proliferation, the PA-Induced Lipogenesis and Inflammation in SZ95 Sebocytes

To assess the interaction of EGF with lipids in regulating sebocyte functions, such as proliferation, lipogenesis and inflammation (**Figure 1B**), we used the human sebaceous gland cell line SZ95, the most accepted *in vitro* human sebocyte model (53). In order to choose a sebum lipid for our studies, which has bio-active properties that may be linked to pathological conditions such as acne, we performed ELISA measurements using supernatants from SZ95 sebocytes treated with sebum lipids such as PA, linoleic acid (LA), oleic acid (OA) and arachidonic acid (AA), which revealed that only PA was a potent inducer of IL6 secretion, a marker for acne-associated sebocyte inflammation (21) (**Supplementary Figure 1**).

SZ95 sebocytes cultured in the presence of EGF exhibited a significantly higher proliferation at 72 and 120 hours compared to cells cultured in the absence of EGF. Importantly, when PA was also added to the culture at the 48 hours timepoint, as described in the Materials and Methods, a tendentious decrease was found in EGF induced proliferation at the 72 and 120 hours timepoints (**Figure 1C**).

Lipid amount in EGF-treated, PA-treated and EGF+PA co-treated sebocytes was determined by neutral/polar lipid ratio 72 hours after PA treatment. Lipid measurements revealed that EGF treatment together with PA led to a decreased amount of intracellular lipids when compared to the lipids detected under PA treatment only (**Figure 1D**).

Measuring IL6 protein levels by ELISA from cell supernatants, EGF was able to increase IL6 secretion from SZ95 sebocytes, which was more pronounced under EGF+PA co-treatment. Interestingly, in the absence of EGF, PA treatment only slightly modified IL6 levels (**Figure 1E**).

These results confirmed previous findings that EGF promoted the proliferation of human sebocytes (42, 55), while the presence of a ubiquitously present lipid, such as PA, induced lipogenesis. Our novel findings that EGF was an inducer of IL6 in sebocytes and that the inflammatory effect of PA was dependent on EGF have set the basis for our genome-wide gene expression studies.

### EGF Promotes IL1 Signaling in SZ95 Sebocytes

**Figure 2:**
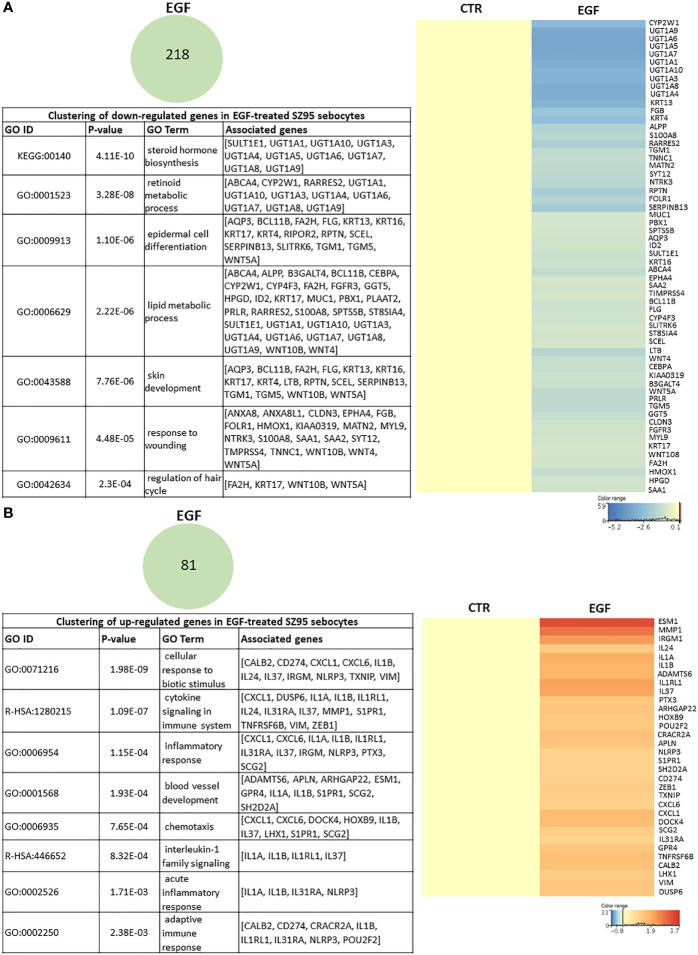
EGF-induced transcriptional changes in SZ95 sebocytes. SZ95 sebocytes were cultured in the presence or absence of EGF. Samples for RNA-seq measurements were harvested at 72 hours of culturing, processed and measured as described in Materials and Methods. **(A)** The 218 transcripts that were significantly down-regulated in SZ95 sebocytes cultured in the presence of EGF (EGF) when compared to cells cultured in the absence of EGF (CTR) were clustered into the groups of steroid hormone biosynthesis, lipid metabolic process, skin development and response to wounding. Heat map display of the down-regulated transcripts contributing to the clusters. Differentially expressed genes were normalized to control and results are expressed as average mean of three samples from subcultures of SZ95 sebocytes. Color intensities reflect the ratios of signal intensities. **(B)** The 81 transcripts that were significantly up-regulated in SZ95 sebocytes cultured in the presence of EGF (EGF) when compared to cells cultured in the absence of EGF (CTR), were clustered into the groups of IL1 signaling, blood vessel development and inflammatory responses. Heat map display of the up-regulated transcripts contributing to the clusters. Differentially expressed genes were normalized to control and results are expressed as average mean of three samples from subcultures of SZ95 sebocytes. Color intensities reflect the ratios of signal intensities as shown.

To identify the EGF-induced transcriptional changes in sebocytes, we compared the gene expression profile of SZ95 sebocytes cultured with or without EGF supplementation in the medium for 72 hours as described in the Materials and Methods. Functional clustering of the 218 down-regulated transcripts resulted in clusters related to steroid, retinoid and lipid metabolism, and to epidermal differentiation (**Figure 2A**), while the up-regulated 81 transcripts were most prominent in the gene clusters involved in IL1 signaling, chemotaxis and blood vessel development (**Figure 2B**).

### Gene Expression Profile of PA and EGF Co-Treated Sebocytes Is Distinct From the Profile of Cells Treated With Either PA or EGF Alone

**Figure 3:**
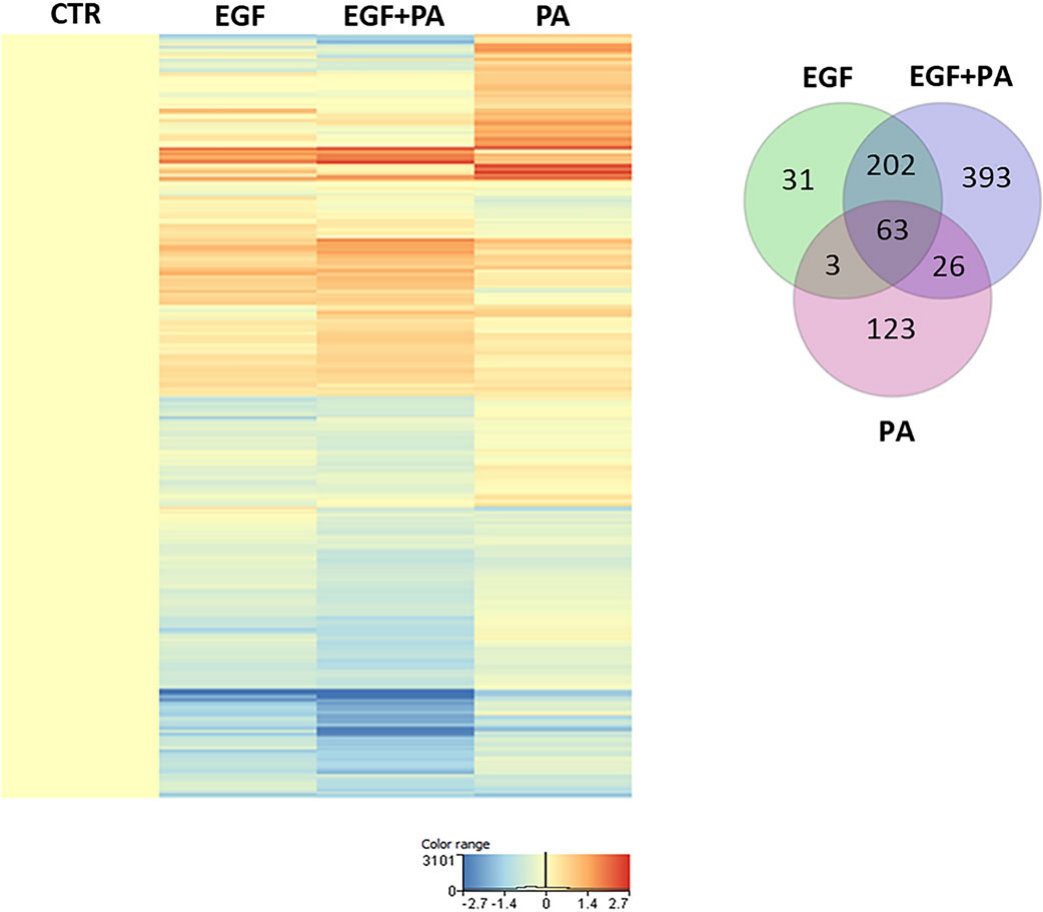
Gene expression profile of EGF-treated, PA-treated and EGF+PA-co-treated sebocytes. Differentially expressed genes shown as a heat map in SZ95 sebocytes treated with PA for 24 hours in the absence (PA) or presence of EGF (EGF+PA), and in SZ95 sebocytes cultured in the presence of EGF (EGF) when compared to cells cultured in the absence of EGF (CTR) as observed by our RNA-seq analysis. Differentially expressed genes were normalized to control and results are expressed as average mean of three samples from subcultures of SZ95 sebocytes. Color intensities reflect the ratios of signal intensities as shown. Venn diagram visualizes the number of genes which are significantly regulated between treatment conditions (EGF (green), EGF+PA (purple) and PA (pink) treated SZ95 sebocytes) 24 hours after PA treatment when compared to SZ95 sebocytes cultured in the absence of EGF (CTR) as observed by our RNA-seq analysis.

To get a deeper insight into how the expression of genes regulated by PA is changed in the presence of EGF, we compared the gene expression profiles of EGF-treated, PA-treated and EGF+PA co-treated SZ95 sebocytes. Generating a heat map with the genes reaching the level of significance at the measured timepoint, 24 hours after PA treatment, showed that while some genes were only regulated in the PA-treated but not in the EGF+PA co-treated sebocytes, a group of genes, which were up-regulated by EGF were further induced when PA was added in combination with EGF. Interestingly, a set of genes was also detected, which were significantly regulated only in the co-treated sebocytes (**Figure 3**).

### PA Treatment in the Absence of EGF Induces Changes Related to Cell Homeostasis

**Figure 4:**
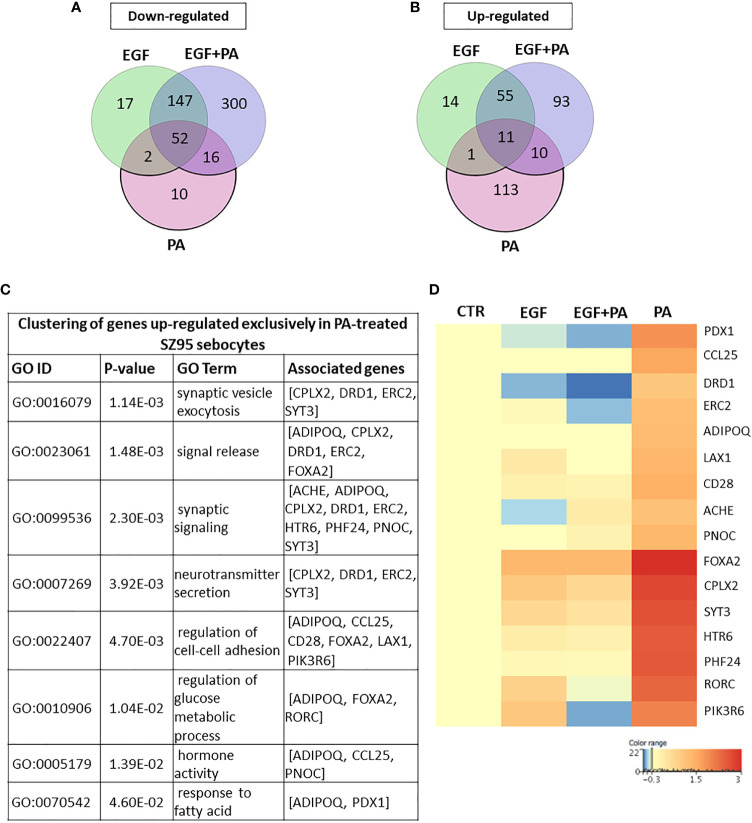
Transcriptional changes induced by PA exclusively when SZ95 sebocytes are cultured without EGF. **(A)** Venn diagram visualizing the number of genes which are significantly down-regulated between treatment conditions (EGF (green), EGF+PA (purple) and PA (pink) treated SZ95 sebocytes) 24 hours after PA treatment when compared to SZ95 sebocytes cultured in the absence of EGF (CTR) as observed by our RNA-seq analysis. Note that the 10 transcripts which were down-regulated only in the PA treated sebocytes could not be grouped into any functional cluster. **(B)** Venn diagram visualizing the number of transcripts which are significantly up-regulated between treatment conditions (EGF (green), EGF+PA (purple) and PA (pink) treated SZ95 sebocytes) 24 hours after PA treatment when compared to SZ95 sebocytes cultured in the absence of EGF (CTR) as observed by our RNA-seq analysis, revealed that 113 transcripts were specific for the PA treatment. **(C)** The 113 transcripts that were up-regulated only in PA (PA *vs*. CTR) but not in EGF+PA co-treated SZ95 sebocytes were functionally categorized into groups related to cell homeostasis. **(D)** Heat map display of the transcripts significantly up-regulated only in PA treated (PA), but not in EGF+PA co-treated (EGF+PA) SZ95 sebocytes that contributed to the clusters. Differentially expressed genes were normalized to control and results are expressed as average mean of three samples from subcultures of SZ95 sebocytes. Color intensities reflect the ratios of signal intensities as shown.

Using Venn diagrams to visualize the genes differentially down- and up-regulated in response to PA, 80 transcripts were found to be down- (**Figure 4A**) and 135 transcripts to be up-regulated (**Figure 4B**) in the PA-treated SZ95 sebocytes when compared to control. While of the 80 down-regulated transcripts only 10, of the 135 up-regulated ones 113 were found to be regulated exclusively in PA treated sebocytes cultured without EGF. After performing clustering, the down-regulated 10 transcripts, could not be grouped into any with functional relevance. However, the 113 transcripts up-regulated only in the PA treated sebocytes cultured without EGF, formed clusters related to cell homeostasis, such as cell adhesion, glucose metabolic processes and hormone activity, synaptic formation, regulation of secretion and fatty acid responses (**Figure 4C**), in which the increased expression of *forkhead box A2 (FOXA2)*, *complexin 2 (CPLX2)*, *RAR related orphan receptor C* (*RORC)*, *phosphoinositide-3-kinase regulatory subunit 6* (*PIK3R6)*, *pancreatic and duodenal homeobox 1* (*PDX1*) and *adiponectin (ADIPOQ)* genes, as displayed in the heat map, could have a potential role (**Figure 4D**).

### PA Treatment Gains Its Potential to Regulate Extracellular Matrix Formation, Lipid Metabolism and Inflammation Related Genes in Sebocytes in the Presence of EGF

**Figure 5:**
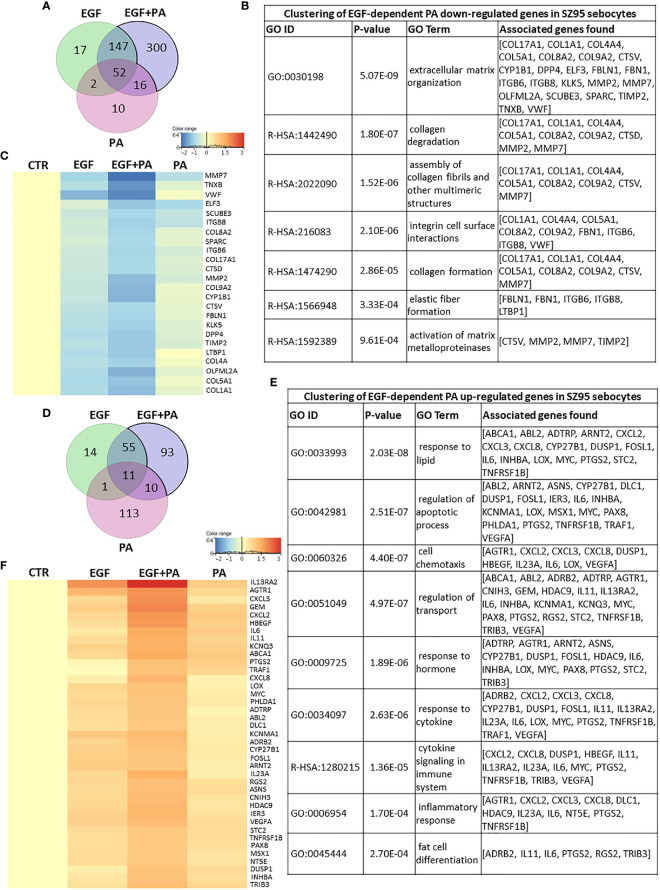
Transcriptional changes induced by PA exclusively when SZ95 sebocytes are cultured in the presence of EGF. **(A)** Venn diagram visualizing that 300 transcripts were significantly down-regulated only in the condition when both EGF and PA was present in the culturing medium of the SZ95 sebocytes as observed by our RNA-seq analysis. **(B)** The 300 transcripts that were down-regulated only in SZ95 sebocytes cultured in the presence of both EGF and PA were functionally categorized into groups of extracellular matrix organization, collagen and elastic fiber formation. **(C)** Heat map display of the ones out of the 300 transcripts with significant down-regulation only in the EGF+PA co-treated sebocytes which were forming clusters. Differentially expressed genes were normalized to control and results are expressed as average mean of three samples from subcultures of SZ95 sebocytes. Color intensities reflect the ratios of signal intensities as shown. **(D)** Venn diagram visualizing that 93 transcripts were significantly up-regulated only when both EGF and PA was present in the culturing medium of the SZ95 sebocytes as observed by our RNA-seq analysis. **(E)** Functional clustering of the 93 transcripts showing significant up-regulation only in SZ95 sebocytes cultured in the presence of both EGF and PA confirmed that PA together with EGF had a complex regulation on genes involved in both lipid metabolism and inflammation. **(F)** Heat map display of the cluster forming 93 differentially expressed transcripts that showed a significant up-regulation only in the EGF+PA co-treated SZ95 sebocytes. Differentially expressed genes were normalized to control and results are expressed as average mean of three samples from subcultures of SZ95 sebocytes. Color intensities reflect the ratios of signal intensities as shown. Note that many of the transcripts had a tendency of up-regulation by EGF and/or by PA treatment, but the co-treatment was necessary to reach the level of significance.

To define the EGF-dependent effects of PA at the level of gene expression regulation, we first addressed the 300 down-regulated transcripts, which were only detected in the EGF+PA co-treated SZ95 sebocytes (**Figure 5A**). Functional clustering revealed that EGF and PA when applied together, decreased the expression of genes involved in modulating the extracellular matrix (**Figure 5B**). The heat map displaying the transcripts which are forming the functional clusters, shows that mostly genes encoding matrix metalloproteinases and collagens are suppressed as a result of the co-treatment (**Figure 5C**).

The Venn diagram, with the 169 transcripts that were significantly up-regulated in the EGF+PA co-treated sebocytes showed that in the case of 93 genes a significant regulation was only detected in the EGF+PA co-treated sebocytes (**Figure 5D**). Analysis of the up-regulated transcripts revealed that the most significant changes were related to genes involved in lipid responses, regulation of apoptotic processes, cytokine signaling and inflammatory responses (**Figure 5E**). Interestingly, displaying the transcripts which contributed to the defined functional clusters in a heat map, revealed that many had a tendency of up-regulation already by EGF and/or by PA treatment, but reached the level of significance only when sebocytes were co-treated with EGF+PA (**Figure 5F**).

### PA Treatment Augments the Inflammatory Effects of EGF at the Level of Gene Expression

Next, we aimed to analyze the expression of the genes which were significantly regulated both in EGF-treated, PA-treated and in the EGF+PA co-treated sebocytes.

**Figure 6:**
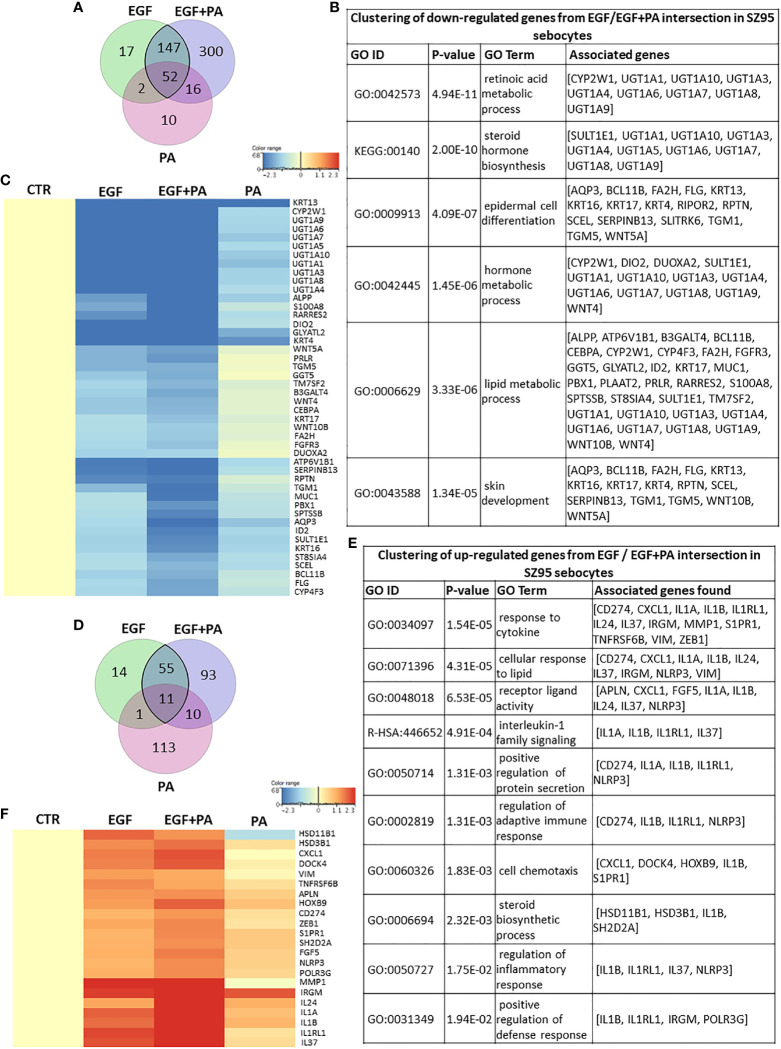
Combined actions of PA and EGF on the gene expression profile of SZ95 sebocytes. **(A)** Venn diagram showing the 199 transcripts which were significantly down-regulated when EGF or EGF+PA was used in combination to treat SZ95 sebocytes, as observed by our RNA-seq analysis. **(B)** Functional clustering of the 199 transcripts showing significant down-regulation in the EGF and the EGF+PA co-treated SZ95 sebocytes revealed that the most significant changes were related to genes involved in hormone and lipid metabolic processes of sebocytes. **(C)** Heat map display of the transcripts which were clustered out of the 199 transcripts with significant down-regulation both in the EGF and the EGF+PA co-treated SZ95 sebocytes. Differentially expressed genes were normalized to control and results are expressed as average mean of three samples from subcultures of SZ95 sebocytes. Color intensities reflect the ratios of signal intensities. **(D)** Venn diagram showing the 66 transcripts which were significantly up-regulated when EGF or EGF+PA was used in combination to treat SZ95 sebocytes, as observed by our RNA-seq analysis. **(E)** Functional clustering of the 66 genes displayed in the Venn diagram defined that EGF alone and in combination with PA regulated immune features of SZ95 sebocytes, notably IL1 signaling. **(F)** Heat map display of the genes which were clustered out of the 66 differentially expressed transcripts. Differentially expressed genes were normalized to control and results are expressed as average mean of three samples from subcultures of SZ95 sebocytes. Color intensities reflect the ratios of signal intensities. Note the interaction between EGF and PA to regulate the expression of the genes which were already significantly regulated either by EGF or by PA.

When analyzing the overlapping 199 down-regulated transcripts displayed in the Venn diagram (**Figure 6A**), the same clusters, such as steroid, retinoid and lipid metabolism, and epidermal proliferation were obtained as from assessing the EGF-induced transcriptional changes (**Figure 6B**), but with a greater number of contributing transcripts (**Figure 6C**).

Functional clustering of the 66 up-regulated transcripts (**Figure 6D**), provided clusters related to immune functions, such as cytokine regulation and chemotaxis, regulation of adaptive immune response and IL1 signaling (**Figure 6E**). Importantly, the heat map presenting the relevant genes which contributed to the clusters further confirmed our previous findings that PA and EGF together might interact in shaping sebocyte functions and augment their inflammatory properties at the level of gene expression regulation (**Figure 6F**).

### Meta-Analysis of Gene Expression Profiles of Acne Samples, EGF-Treated and EGF+PA- Co-Treated SZ95 Sebocytes Suggests That PA Together With EGF May Play a Role in Inducing Inflammation Under (Patho)physiological Conditions

To provide further biological relevance for our studies, a meta-analysis was performed using available gene expression data of acne whole tissue samples (56).

**Figure 7:**
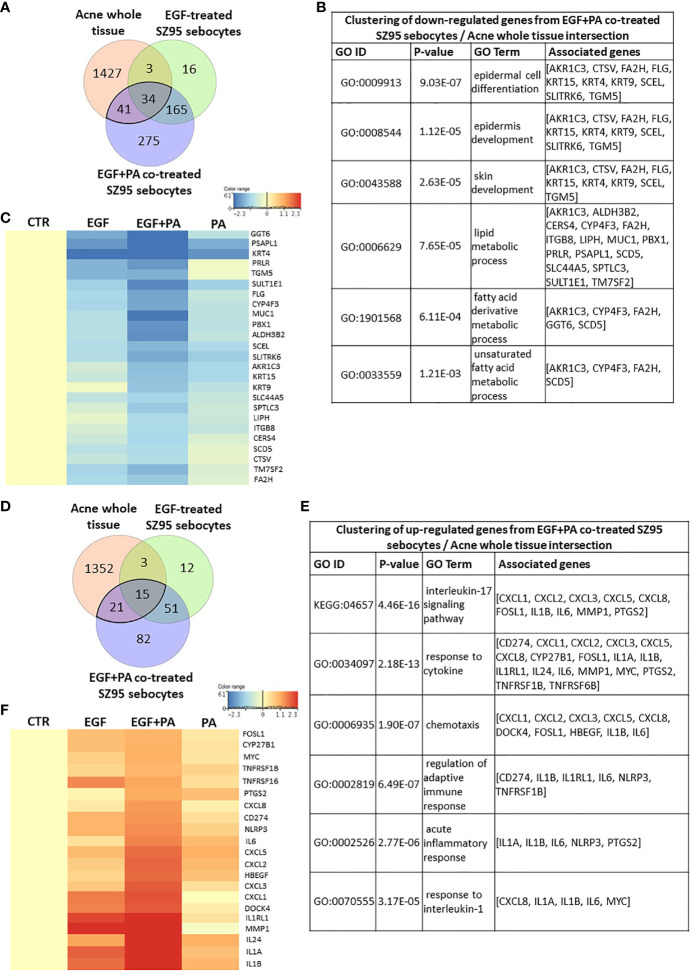
Meta-analysis using gene expression profiles from acne samples, EGF-treated and EGF+PA- co-treated SZ95 sebocytes. **(A)** Venn diagram visualizing the number of transcripts that were significantly down-regulated in acne whole tissue samples from the available gene expression profiles of Kelhala HL et al. (NCBI GEO accession number: GSE5379) (56) and in EGF and EGF+PA co-treated SZ95 sebocytes. **(B)** Functional clustering of the 75 (41 + 34) transcripts that were significantly down-regulated both in the EGF+PA co-treated SZ95 sebocytes and in acne whole tissue samples (56) revealed that the most significant changes were related to genes involved in skin development and fatty acid metabolic process. **(C)** Heat map display of the genes which were clustered out of the 75 significantly down-regulated transcripts. Differentially expressed genes were normalized to control and results are expressed as average mean of three samples from subcultures of SZ95 sebocytes. Color intensities reflect the ratios of signal intensities as shown. **(D)** Venn diagram visualizing the number of transcripts that were significantly up-regulated in acne whole tissue samples from the available gene expression profiles of Kelhala et al. (56) and in EGF and EGF+PA co-treated SZ95 sebocytes. **(E)** Functional clustering of the 36 transcripts that were significantly up-regulated both in the EGF and EGF+PA co-treated SZ95 sebocytes and in acne whole tissue samples (56) revealed that EGF in combination with PA may contribute to the (patho)physiologically relevant IL1 and IL17 signaling in SZ95 sebocytes. **(F)** Fold change value based hierarchical clustering of the cluster forming genes, showing their expression levels in the PA, EGF and EGF+PA co-treated SZ95 sebocytes, that are significantly up-regulated in the EGF and EGF+PA co-treated SZ95 sebocytes when compared to untreated cells at 24 hours and in the available gene expression profiles of acne whole tissue samples (56). Differentially expressed genes were normalized to control and results are expressed as average mean of three samples from subcultures of SZ95 sebocytes. Color intensities reflect the ratios of signal intensities as shown. Note that the majority of the identified genes, showing a prominent expression in SZ95 sebocytes treated with PA in combination with EGF (such as *CXCL8*, *IL1A*, *IL1B*, *IL6*, *NLRP3* and *PTGS2*), are pivotal in the pathogenesis of acne.

Using a Venn diagram to visualize the down-regulated transcripts in acne samples compared to healthy ones and in the EGF-treated and EGF+PA-co-treated SZ95 sebocytes compared to untreated controls, a possible contribution of 34 significantly altered transcripts from EGF-treated and an additional 41 transcripts from the EGF+PA-co-treated sebocytes to acne pathogenesis could be detected (**Figure 7A**), with a possible role in the differentiation and lipid metabolism of sebocytes (**Figure 7B**). The heat map revealed that the involved genes had the highest change in their expression levels when sebocytes were co-treated with EGF+PA (**Figure 7C**).

When assessing the up-regulated transcripts a possible contribution of 15 significantly up-regulated transcripts from EGF-treated and an additional 21 transcripts from the EGF+PA-co-treated sebocytes could be detected (**Figure 7D**). Clustering the identified genes, showed that in the EGF+PA-co-treated sebocytes not only IL1 but also IL17 signaling, characteristic both for SG-rich healthy skin and for acne-involved skin, was induced (**Figure 7E**). Generation of a heat map with the transcripts contributing to the clusters, confirmed that EGF when applied with PA may have more prominent inflammatory effects, as revealed by the enhanced expression of IL1 signaling related genes, compared with conditions where sebocytes were only treated with EGF or PA. Interestingly and in line with our previous findings, genes such as *C-X-C motif chemokine ligand 8* (*CXCL8)* and *prostaglandin-endoperoxidase synthase 2* (*PTGS2)* were only induced significantly when sebocytes were co-treated with EGF+PA (**Figure 7F**). Importantly, despite the low number of the overlapping genes, the majority of the identified transcripts (such as *CXCL8, IL1B, IL6, NLR family pyrin domain containing 3* [*NLRP3*] and *PTGS2)* are also pivotal in acne pathogenesis, suggesting that EGF- and PA-induced signaling in sebocytes may have a (patho)physiological relevance.

## Discussion

Using an unbiased system-based approach of RNA-sequencing of SZ95 sebocytes, the best characterized sebaceous *in vitro* model, to reveal the effects of EGF, PA or their combination, we provide evidence that EGF and PA may contribute together to sebocyte functions both under physiological conditions as well as in disease settings, such as acne.

Sebocyte inflammatory signaling, marked by increased expression of cytokines IL6, IL8, IL1B and lipid-metabolizing enzymes, such as 5-lipoxygenase (5LOX) or cyclooxygenase 2 (COX2 or PTGS2), was shown to be increased in acne (21, 57), however prominent immune and barrier differences were observed already in the healthy skin rich in SGs compared to that with less SGs (11, 58). The latter finding, therefore, raised the possibility that even under physiological conditions, SG may determine basic immune features and/or contribute to the altered microbiome directly with their lipids and proteins (59), where first explanation of the mechanisms have currently been provided (17, 58). Our findings that EGF alone and in combination with PA induces the expression of immune-related genes and pathways in sebocytes, add further details and provide an example on how physiological signals may facilitate SGs to become immunologically active. However, limitations might arise from our *in vitro* settings such as using a single cell line, the number of the analyzed replicates and the possibility that sebocytes with their active enzymatic machinery, could modify PA and, therefore, the produced derivatives may contribute to further processes (13).

Important result of our studies was the finding that EGF alone was able to promote IL1 signaling at the level of gene expression, which complemented previous studies using cetuximab - an EGFR inhibitor - in SZ95 sebocytes, where inhibition of EGF signaling increased the expression levels of inflammatory cytokines IL6, IL8 and TNFA, but not of IL1A. Since IL1A is a key cytokine to modulate not only inflammation, but also follicular keratinocyte proliferation and abnormal differentiation, leading to comedogenesis, the initial step in the development of acne, which is missing in cetuximab-induced papulopustular eruptions (50), our results provide interesting start points for further studies to assess the role of EGF in the pathogenesis of diseases with follicular obstruction. Interestingly, the findings that in the EGF-treated sebocytes, IL6 was expressed at higher mRNA and secreted protein levels when compared to the cells cultured in the absence of EGF, suggests that the inhibition and the absence of EGF signaling should be addressed separately. Moreover, studies on sebocytes should be performed and evaluated also with a care on the presence of EGF in the culture medium. As revealed by our results, the inflammatory properties and the proliferation of the cells are already altered at the level of gene expression when sebocytes are cultured with EGF, just as the lipid profile and the metabolism of retinoids and steroids may be affected as well (24, 26, 60).

One of the most interesting findings is that PA - a major component of sebum ubiquitously synthetized and secreted from sebocytes - is a regulator of sebocyte homeostasis, and its effect is more pronounced in the presence of EGF, since it suggests a so far uncharacterized role for sebum in contributing to SG homeostasis. Furthermore, our results suggests that SGs could even contribute to the integrity of the entire pilosebaceous unit. Although their possible effect on vascularization has already been proposed with the detection of vascular endothelial growth factor (VEGF) in SGs (61), SGs may also change their production of connective tissue elements and of enzymes involved in modifying the extracellular matrix (62), which findings definitely call for further studies.

Our further results that in the presence of EGF, PA could also have an immunologically active role, and the results that induction of a group of genes was only reaching the level of significance when both PA and EGF were present, suggest that these agents could promote each other and therefore both EGF and PA (regarding levels and signaling pathways) could be of therapeutic relevance. Moreover, the prominent induction of genes, such as *PTGS2, CXCL8, IL6*, *IL1B, matrix metalloproteinase 1* (*MMP1*) and *NLRP3*, even raised the possibility that EGF and PA may be involved in the pathogenesis of acne. Based on these findings it is reasonable to speculate that the altered ratio of sebum components (2) and the increased amount of sebum - and with that the increased PA levels - might have regulatory and inflammatory roles in the manifestation of this disease, however only when other factors, that are yet to be identified in more details, are present (63).

**Figure 8:**
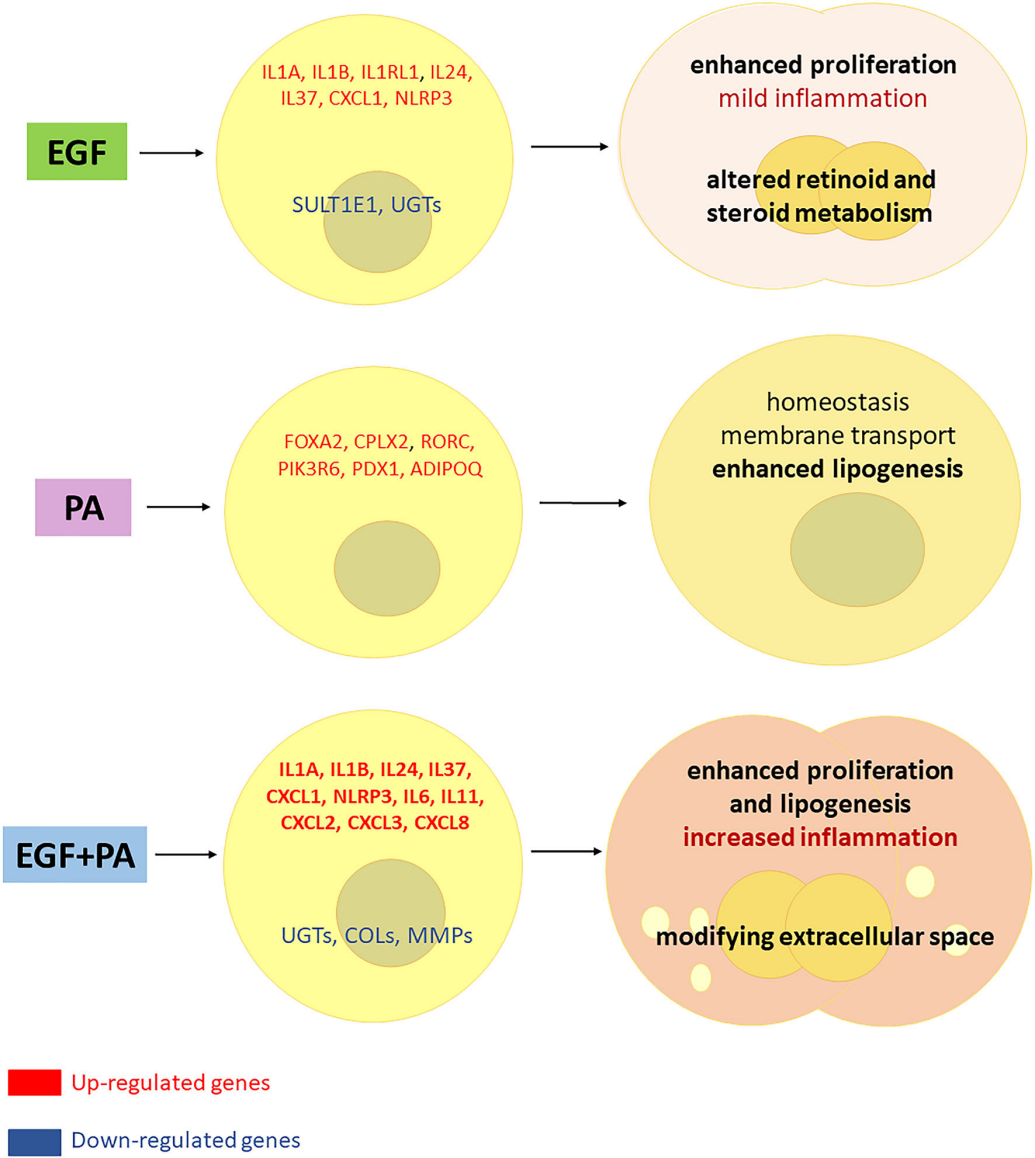
Interaction between EGF and PA to modulate cell proliferation, lipogenesis and inflammatory responses in sebocytes. Based on our results, EGF induces proliferation and inflammatory pathways in sebocytes while down regulates the expression of genes involved in steroid and retinoid metabolism. PA treatment regulates lipogenesis and induces genes related to cell homeostasis in human sebocytes. When both EGF and PA is present, sebocytes exert an increased proliferation, lipogenesis and a prominent up-regulation of inflammatory genes, while genes involved in the extracellular matrix formation are down-regulated, which may have (patho)physiological relevance in shaping the inflammatory dermal environment.

Altogether our results confirmed previous findings that EGF is a stimulator of sebocyte proliferation, and provided new results supporting its inflammatory effect by IL1 pathway induction. We also extended our knowledge on the role of PA in sebocytes and revealed that PA is primarily involved in cell homeostasis, but can become a potential inflammatory agent in the presence of EGF. The findings that sebocytes treated with both EGF and PA, while conserving their enhanced proliferation and increased lipogenesis, also acquired a prominent inflammatory gene expression profile may have further implications in understanding acne development (**Figure 8**). Our results are of great importance also in regards of promoting a system-based approach in basic research, pointing on the need to also explore combined effects of relevant stimuli, which may bring us closer in understanding the complexity of sebaceous immunobiology and acne development.

## Data Availability Statement

The datasets presented in this study can be found in online repositories. The names of the repository/repositories and accession number(s) can be found in the article/**Supplementary Material**.

## Ethics Statement

The studies involving human participants were reviewed and approved by Regional and Institutional Ethics Committee, University of Debrecen, Hungary. The patients/participants provided their written informed consent to participate in this study.

## Author Contributions

DT, AG, FF and DK designed the experimental protocol. FF and AG performed the experiments. SP, DT, FF, AG and DK performed the analyses of RNA-Seq data. EJ and SP performed the statistical analyses. DT, DK, FF, AG, KD, AS and CZ interpreted the data. DT, DK and CZ wrote the manuscript. All authors contributed to the article and approved the submitted version.

## Funding

This research was supported by the Hungarian National Research, Development and Innovation Fund (FK-132296 and K-128250) to DT and to AS, the New National Excellence Program of the Ministry of Human Capacities and the János Bolyai research scholarship of the Hungarian Academy of Sciences to DT, the GINOP-2.3.2-15-2016-00005 (AG, KD, DK and DT) project and the Doctoral School of Health Sciences, University of Debrecen (FF). The funders had no role in study design, data collection and analysis, decision to publish, or preparation of the manuscript.

## Conflict of Interest

The authors declare that the research was conducted in the absence of any commercial or financial relationships that could be construed as a potential conflict of interest.
