# Supplementary material for: Epidermal Growth Factor Modulates Palmitic Acid-Induced Inflammatory and Lipid Signaling Pathways in SZ95 Sebocytes
Source: Front Immunol. 2021 May 6;12:600017. doi: 10.3389/fimmu.2021.600017 (PMC8134683; doi:10.3389/fimmu.2021.600017)
Supplement: Supplementary file 1 [file DataSheet_1.pdf]

Supplementary Material

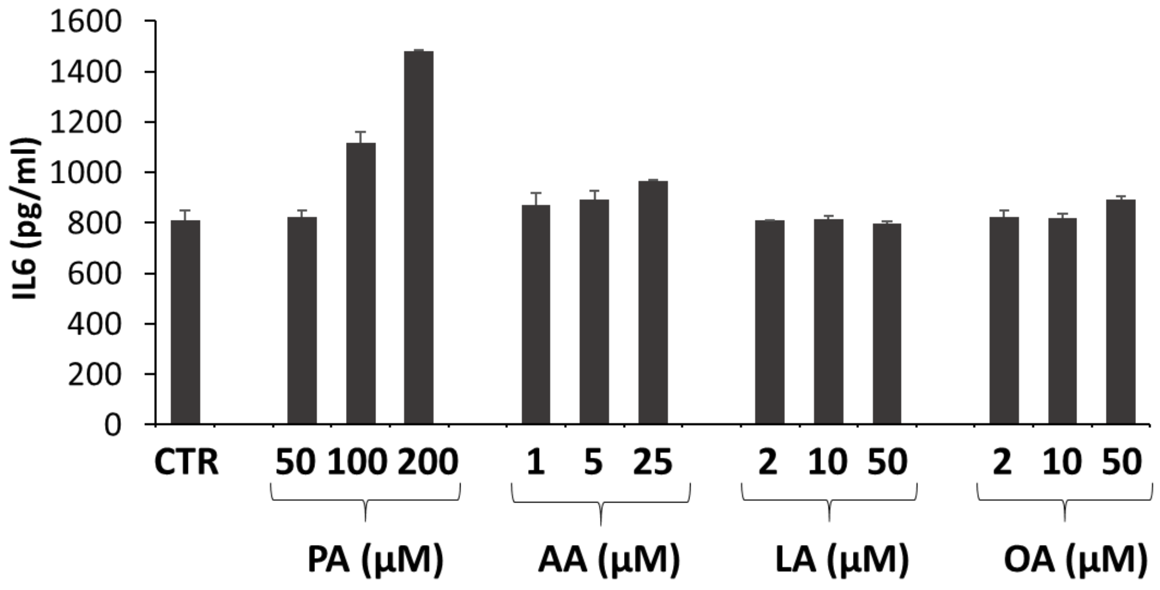

Supplementary Figure 1. Levels of IL6 protein measured by ELISA as described in the Materials and Methods from supernatants of SZ95 sebocytes cultured in the presence of EGF and treated with various sebum lipids such as palmitic acid (PA), arachidonic acid (AA) and linoleic acid (LA) or with oleic acid (OA) in increasing doses for 24 hours.

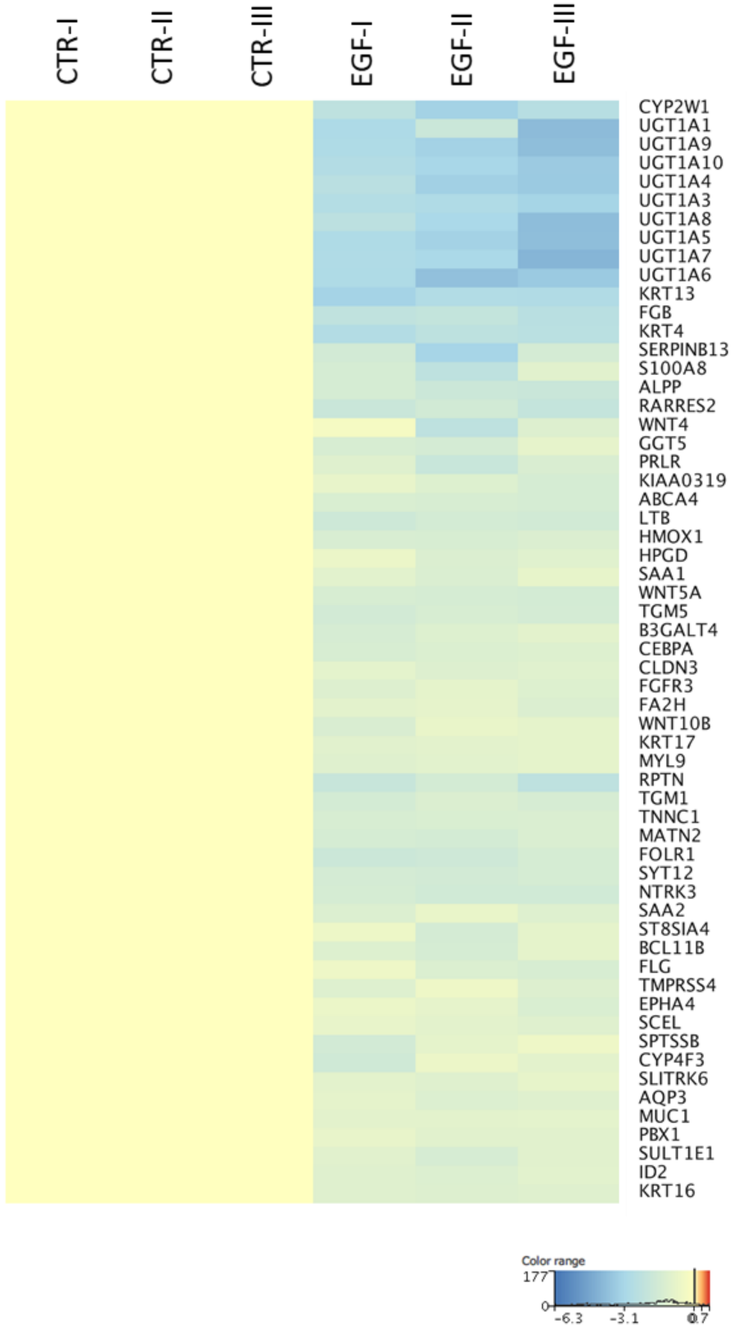

Supplementary Figure 2. Heat map displaying the replicates separately to support the heat map in Figure 2A, which shows mean average values. Color intensities reflect the ratios of signal intensities as shown.

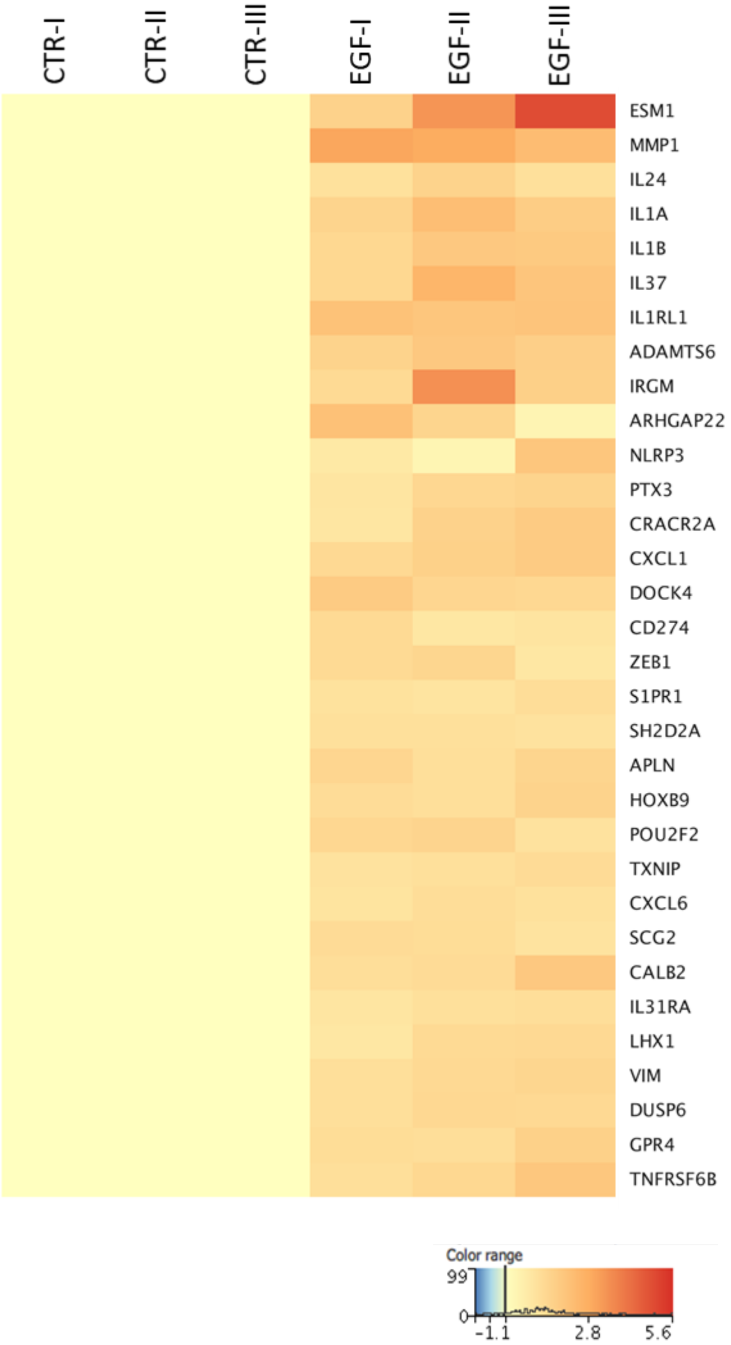

Supplementary Figure 3. Heat map displaying the replicates separately to support the heat map in Figure 2B, which shows mean average values. Color intensities reflect the ratios of signal intensities as shown.

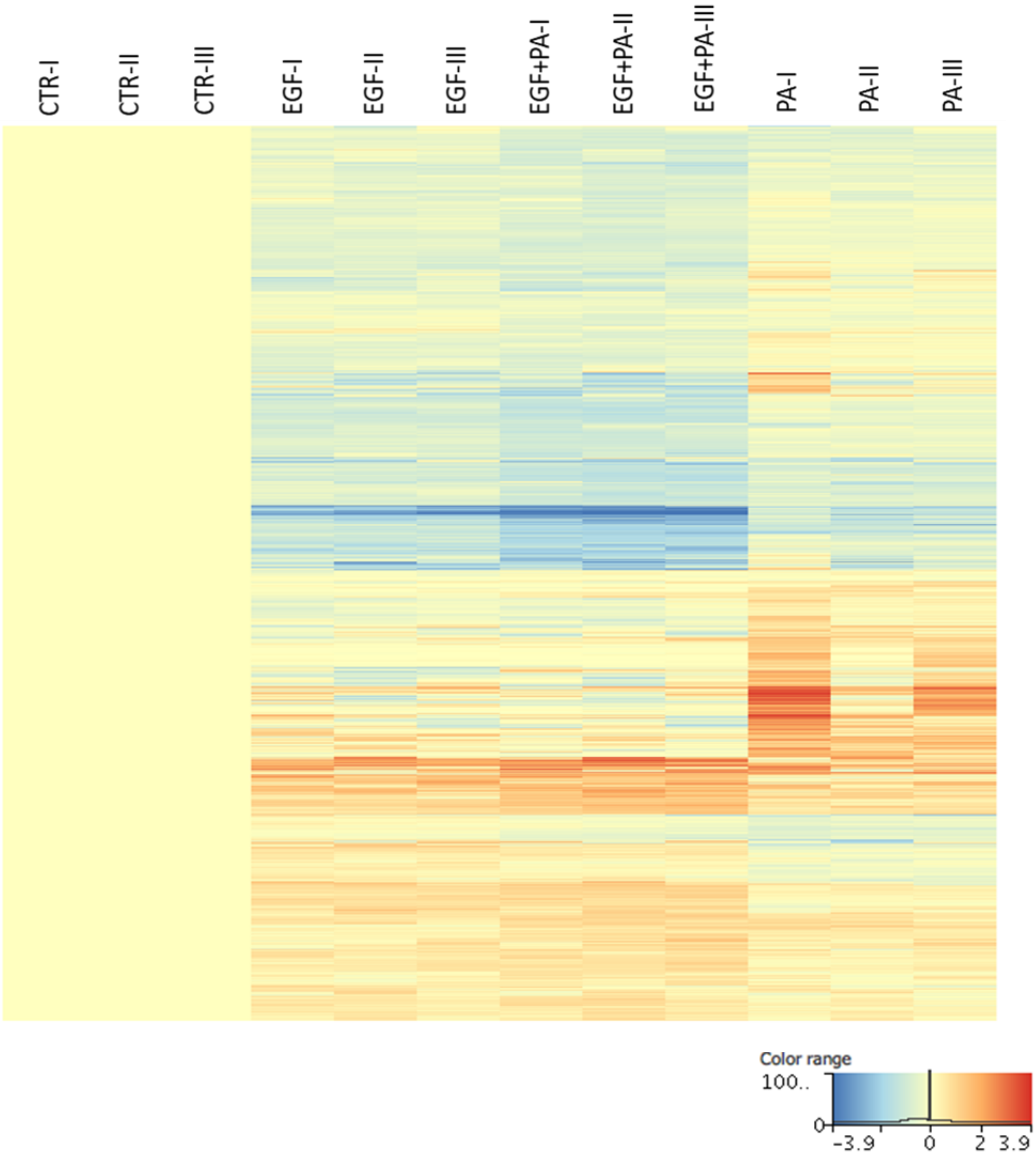

Supplementary Figure 4. Heat map displaying the replicates separately to support the heat map in Figure 3, which shows mean average values. Color intensities reflect the ratios of signal intensities as shown.

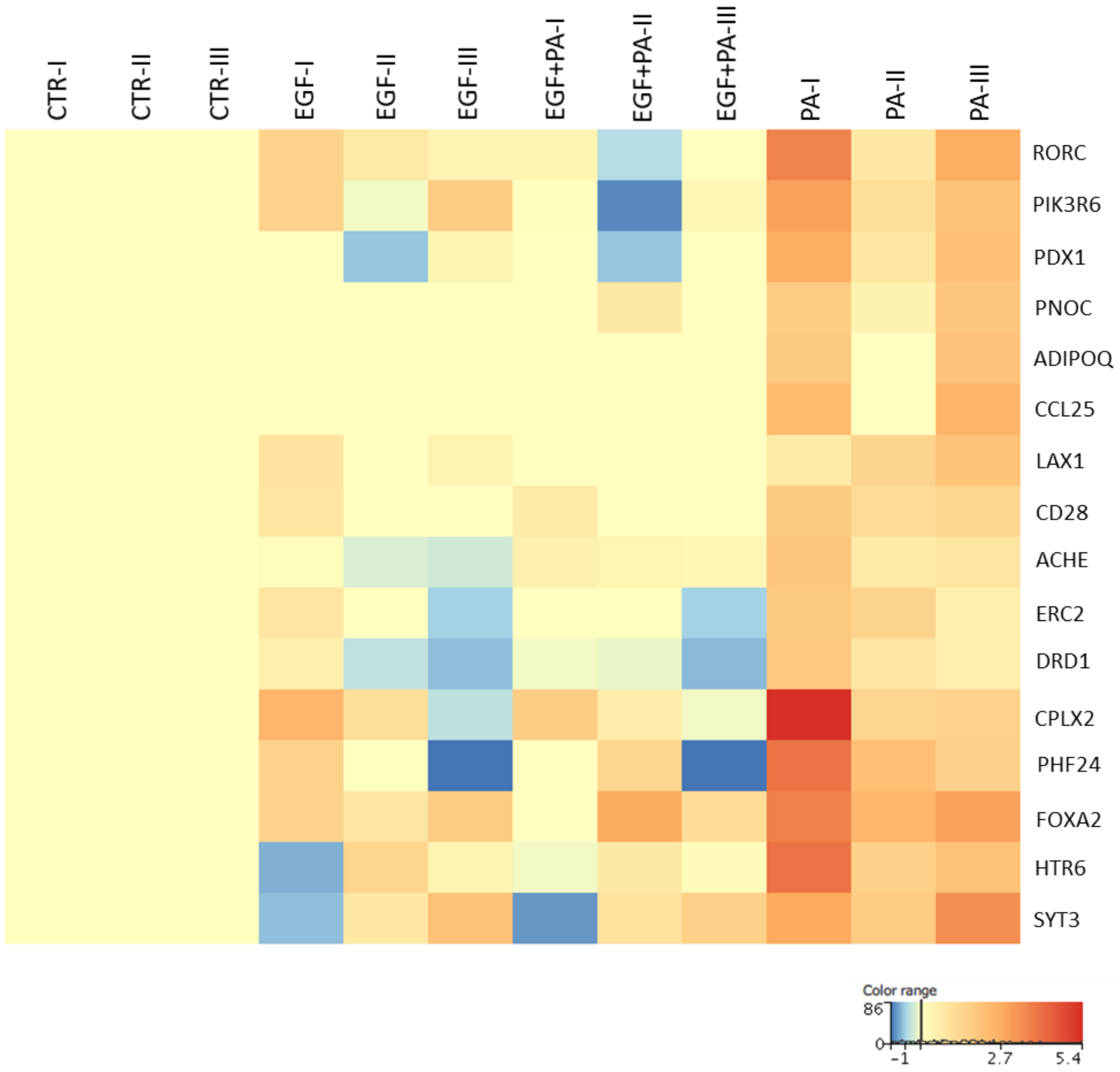

Supplementary Figure 5. Heat map displaying the replicates separately to support the heat map in Figure 4D, which shows mean average values. Color intensities reflect the ratios of signal intensities as shown.

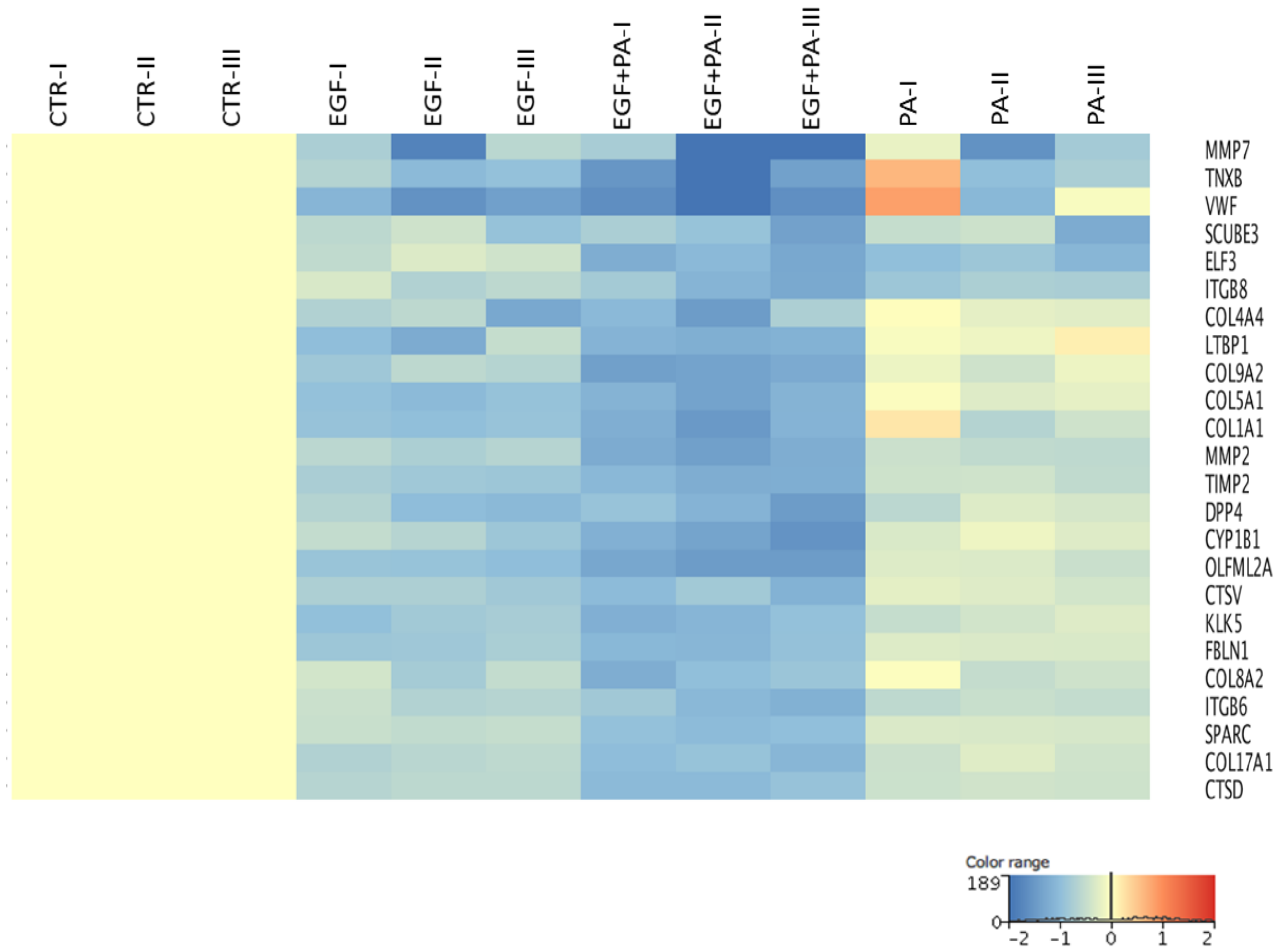

Supplementary Figure 6. Heat map displaying the replicates separately to support the heat map in Figure 5C, which shows mean average values. Color intensities reflect the ratios of signal intensities as shown.

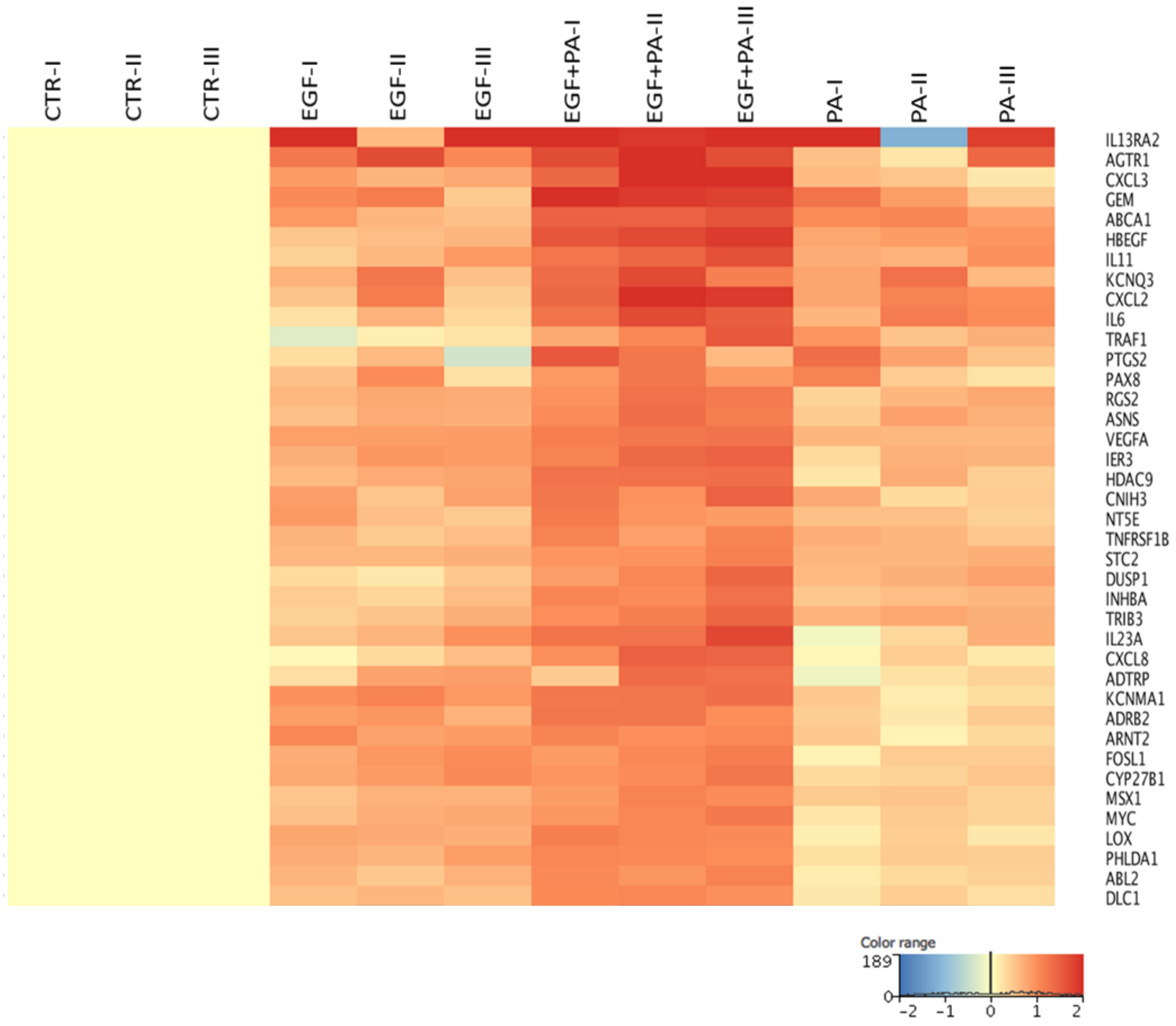

Supplementary Figure 7. Heat map displaying the replicates separately to support the heat map in Figure 5F, which shows mean average values. Color intensities reflect the ratios of signal intensities as shown.

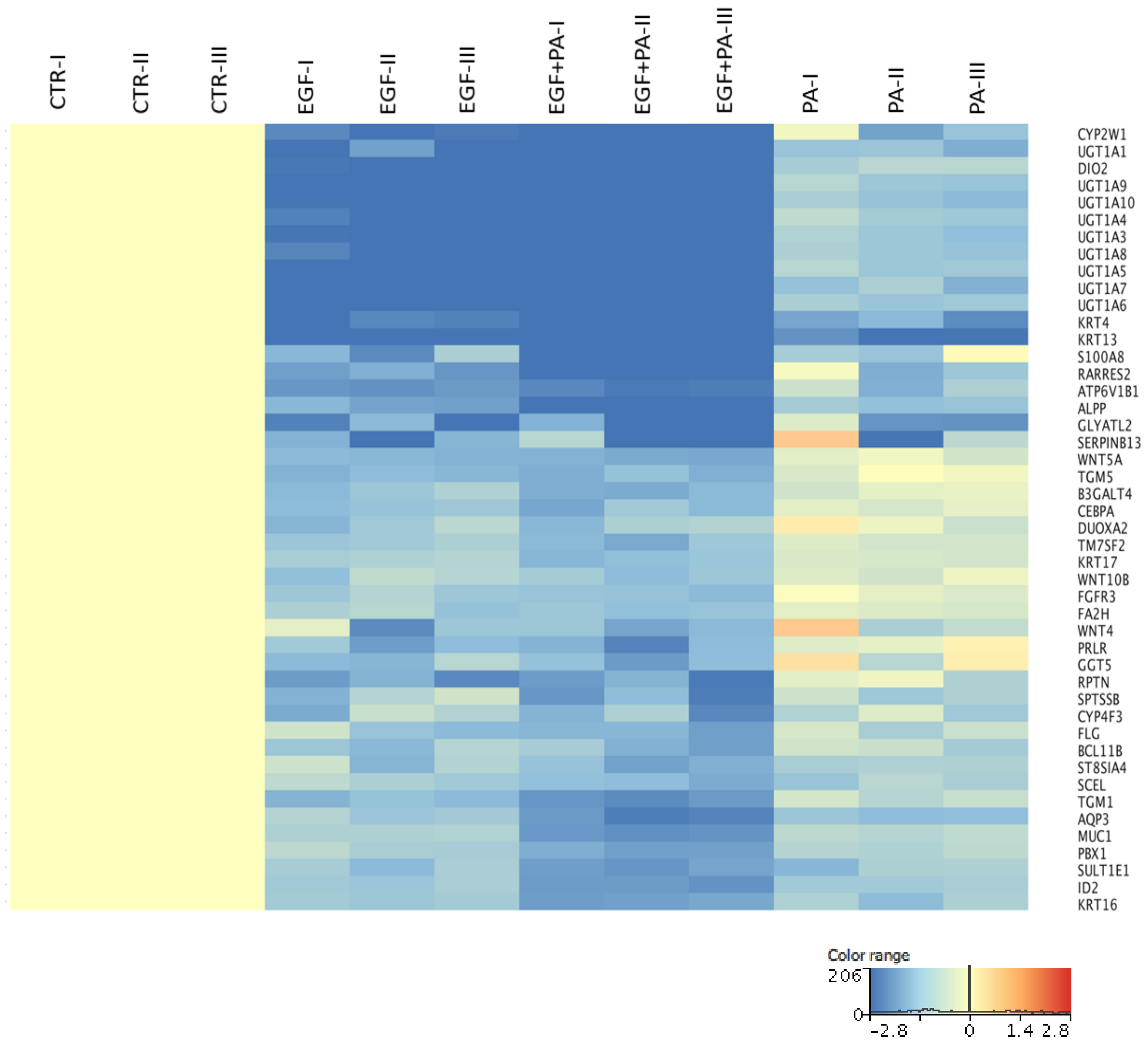

Supplementary Figure 8. Heat map displaying the replicates separately to support the heat map in Figure 6C, which shows mean average values. Color intensities reflect the ratios of signal intensities as shown.

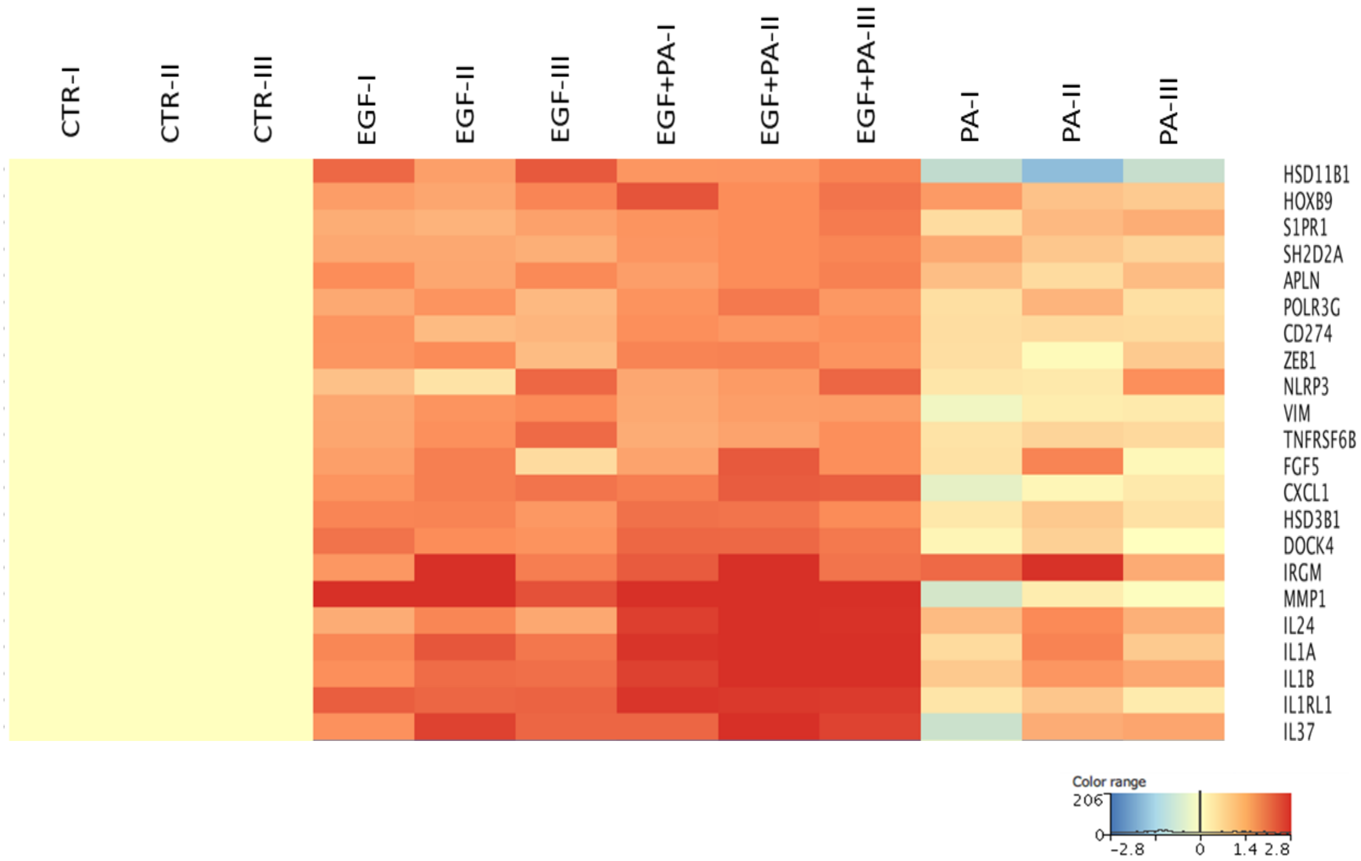

Supplementary Figure 9. Heat map displaying the replicates separately to support the heat map in Figure 6F, which shows mean average values. Color intensities reflect the ratios of signal intensities as shown.

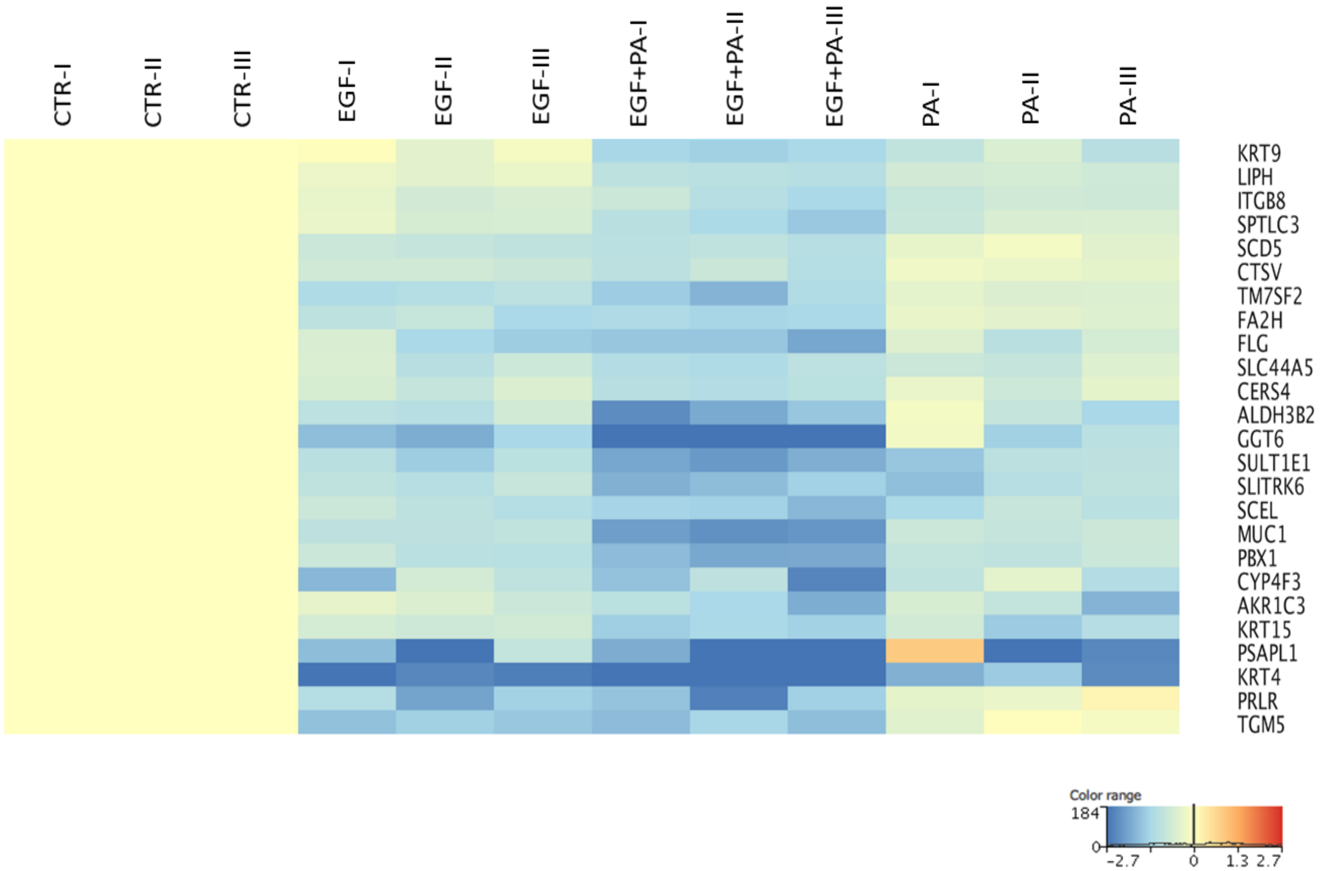

Supplementary Figure 10. Heat map displaying the replicates separately to support the heat map in Figure 7C, which shows mean average values. Color intensities reflect the ratios of signal intensities as shown.

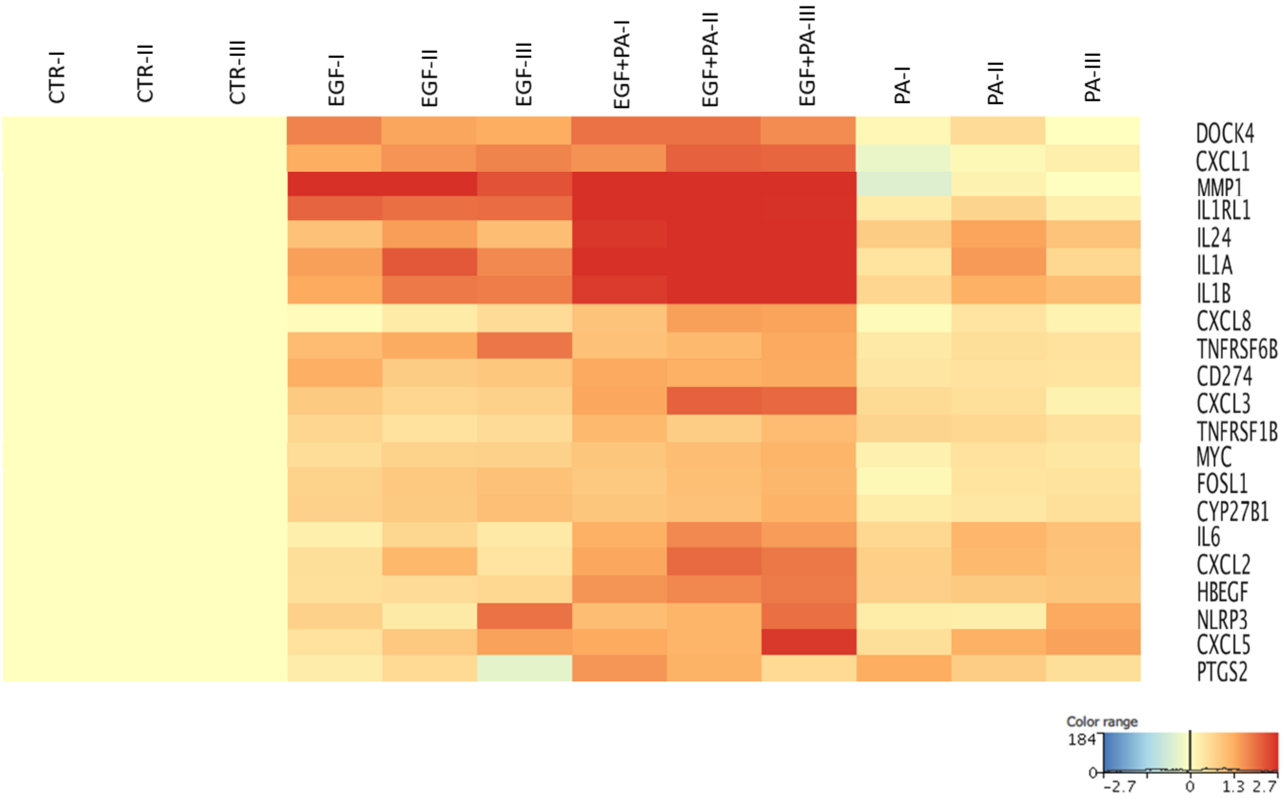

Supplementary Figure 11. Heat map displaying the replicates separately to support the heat map in Figure 7F, which shows mean average values. Color intensities reflect the ratios of signal intensities as shown.

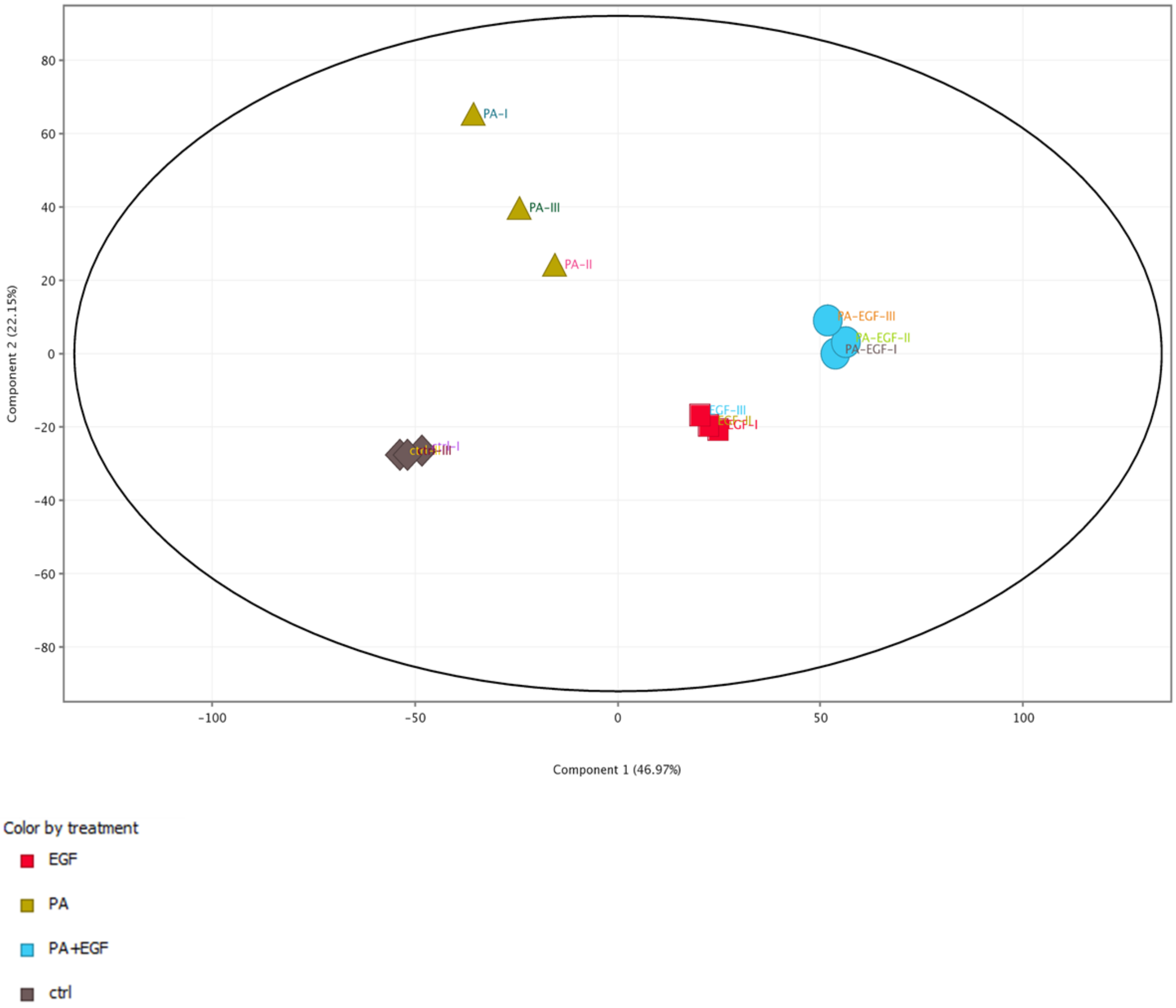

Supplementary Figure 12. Principal component analysis (PCA) plot generated using the normalized gene expression values of EGF (red), PA (yellow), EGF+PA (blue) treated and CTR (black) SZ95 sebocytes. Each dot represents one sample and each color represents the type of treatment. PCA plot shows the gene expression variances between the four different treatments and also between the biological replicates.
